# Prosocial behaviour and happiness among children aged 6 to 11 years in Canada

**DOI:** 10.24095/hpcdp.46.2.03

**Published:** 2026-02

**Authors:** Colin A. Capaldi, Danielle Lemaire, Katrina R. Abela, Laura L. Ooi, Melanie Varin

**Affiliations:** 1 Centre for Surveillance and Applied Research, Public Health Agency of Canada, Ottawa, Ontario, Canada; 2 Offord Centre for Child Studies, McMaster University, Hamilton, Ontario, Canada

**Keywords:** altruism, happiness, child, sociodemographic factors, trends, public health surveillance

## Abstract

A gap in Canadian public health surveillance is the monitoring of childhood positive mental health (PMH). We used available data from the first six cycles of the Canadian Health Measures Survey to examine how two potential PMH indicators are distributed across time and populations of children aged 6 to 11 years. The prevalence of normative parent-rated prosocial behaviour and perceived happiness was high and relatively stable across time. Normative parent-rated prosocial behaviour was more common among females (vs. males) and 8- to 9-year-olds (vs. 6- to 7-year-olds), while perceived happiness was higher among 6- to 7-year-olds (vs. 10- to 11-year-olds).

**Highlights**

- The vast majority of children had normative levels of prosocial behaviour (i.e. scoring 8+ on the prosocial subscale of the parent-rated Strengths and Difficulties Questionnaire)— 89.6% in 2007–2009 to 90.2% in 2014–2015.
- Around nine in ten children were described as being usually “happy and interested in life”—92.0% in 2007–2009 to 91.1% in 2018–2019.
- The prevalence of normative parent- rated prosocial behaviour was higher among females (93.8%) than males (83.3%) and children aged 8 to 9 (89.8%) than 6 to 7 (85.6%) years.
- The prevalence of perceived happiness was higher among children aged 6 to 7 (94.3%) than 10 to 11 (88.9%) years.

## Introduction

The Public Health Agency of Canada monitors positive mental health (PMH) outcomes and their determinants, guided by the Positive Mental Health Surveillance Indicator Framework (PMHSIF).^1^ The importance of taking life course stages into account (i.e. childhood [0 to 11 years], youth [12 to 17 years], adulthood [18+ years]) was identified during the initial PMHSIF development.^2^ Adult and youth versions of the PMHSIF have been released and updated multiple times,^1^ but a child version remains in development. To begin to fill that gap, a scoping review was conducted to understand how PMH is conceptualized in childhood, with hedonic/emotional well-being, psychological well-being, social well-being and social emotional learning/positive development emerging as key aspects of child PMH.^3^

The next step for surveillance is to identify and examine potential indicators of child PMH. The Canadian Health Measures Survey (CHMS) has frequently measured two PMH outcomes among children: prosocial behaviour and happiness. Prosocial behaviours are voluntary actions meant to benefit others—including helping, sharing and comforting^4^—and can be considered part of social emotional learning/positive development (i.e. social awareness, relationship skills, social competence).^5^, ^6^ Happiness definitions have varied across time and cultures,^7^ but it is commonly conceptualized by researchers as a core element of hedonic/emotional well-being (with pleasure, enjoyment or life satisfaction).^8^

Despite these outcomes being included in the earliest CHMS cycles,^9^,^10^ understanding of their epidemiology among Canadian children is limited. To gain insight into what indicators based on these measures would tell us about child PMH if they were used for surveillance, our objectives were to investigate whether levels of happiness and prosocial behaviour among Canadian children (1) vary over time, (2)differ between sociodemographic groups, and (3) are associated with each other.

## Methods

***Data***

The CHMS is a repeated cross-sectional survey by Statistics Canada that collects representative data on the health of people in Canada through questionnaires and direct health measures. We used shared data from the household questionnaires of the first six cycles,^9^-^14^ which were administered via personal interview in the respondent’s home from 2007–2019 (cycle dates are reported in Table 1). The target population was 3 to 79 years of age in cycles 2–6, and 6 to 79 years in cycle 1. Full-time Canadian Forces members, and those living in institutions, on reserves or other Indigenous settlements, and in specific remote regions were not part of the target population, nor were those living in the territories from cycle 3 onward. A multistage sampling strategy is used for the CHMS, where collection sites are selected from regions, followed by a random sample of dwellings and then sampling individuals within households. We restricted our analyses to 6- to 11-year-olds (*N*s = 1081, 1076, 1024, 1032, 1040 and 1011 for cycles 1–6, respectively).

**Table 1:** Comparing normative parent-rated prosocial behaviour and high perceived happiness over time, overall and stratified by sex, children aged 6 to 11 years old, CHMS, 2007–2015/2019, Canada

Normative parent-rated prosocial behaviour							
Category	Cycle 1 Mar 2007 – Feb 2009 Weighted % (95% CI)	Cycle 2 Aug 2009 – Nov 2011 Weighted % (95% CI)	Cycle 3 Jan 2012 – Dec 2013 Weighted % (95% CI)	Cycle 4 Jan 2014 – Dec 2015 Weighted % (95% CI)	Cycle 2 vs. Cycle 1 OR (95% CI)	Cycle 3 vs. Cycle 1 OR (95% CI)	Cycle 4 vs. Cycle 1 OR (95% CI)
Overall	89.6 (86.5–92.8)	86.0 (81.9–90.0)	88.1 (83.2–93.0)	90.2 (87.7–92.8)	0.71 (0.44–1.13)	0.86 (0.47–1.58)	1.07 (0.70–1.64)
Female	93.7 (91.3–96.0)	94.6 (92.2–97.0)	93.4 (90.5–96.3)	93.6 (91.0–96.3)	1.15 (0.60–2.32)	0.96 (0.52–1.77)	0.97 (0.56–1.77)
Male	85.8 (79.3–92.2)	77.9 (70.4–85.3)	83.1 (74.7–91.5)	87.0 (81.8–92.2)	0.58 (0.29–1.17)	0.82 (0.35–1.89)	1.11 (0.55–2.25)

**Table 1b:** Comparing normative parent-rated prosocial behaviour and high perceived happiness over time, overall and stratified by sex, children aged 6 to 11 years old, CHMS, 2007–2015/2019, Canada (continued)

High perceived happiness							
Category	Cycle 1 Mar 2007 – Feb 2009 Weighted % (95% CI)	Cycle 2 Aug 2009 – Nov 2011 Weighted % (95% CI)	Cycle 5 Jan 2016 – Dec 2017 Weighted % (95% CI)	Cycle 6 Jan 2018 – Dec 2019 Weighted % (95% CI)	Cycle 2 vs. Cycle 1 OR (95% CI)	Cycle 5 vs. Cycle 1 OR (95% CI)	Cycle 6 vs. Cycle 1 OR (95% CI)
Overall	92.0 (90.0–93.1)	92.2 (89.5–94.8)	91.3 (87.7–94.8)	91.1 (87.0–95.3)	1.02 (0.68–1.53)	0.01 (0.57–1.43)	0.89 (0.52–1.53)
Female	92.7 (90.3–95.1)	94.9 (92.2–97.6)	89.4 (84.2–94.6)	93.7 (90.2–97.2)	1.46 (0.71–3.00)	0.66 (0.35–1.25)	1.17 (0.59–2.30)
Male	91.5 (89.3–93.2)	89.6 (85.8–93.3)	93.0 (90.0–96.1)	88.7 (81.2–96.1)	0.82 (0.51–1.30)	0.73 (0.74–2.17)	0.71 (0.32–1.75)

**Source:** Canadian Health Measures Survey. 

**Abbreviations: **CI, confidence interval; OR, odds ratio. 

**Notes:** All estimates are weighted. After excluding respondents with missing responses on any of the items in the prosocial behaviour subscale, the combined sample sizes were 4196, 2097, and 2099 for the overall, female and male analyses involving prosocial behaviour, respectively. After excluding respondents with missing responses on the happiness item, the combined sample sizes were 4205, 2091, and 2114 for the overall, female and male analyses involving happiness, respectively. Estimates may differ slightly from those obtained when analyzing individual cycle data due to the modified weights that we used when combining Canadian Health Measures Survey data from multiple cycles. The degrees of freedom was 46. 

***Measures***

Prosocial behaviour was measured in cycles 1–4 (2007–2015) using five items from the prosocial behaviour subscale of the Strengths and Difficulties Questionnaire (SDQ).^15^ The SDQ has been validated for Canadian children and youth, with evidence provided for its factorial validity, internal consistency, and measurement invariance across sex, age, and language.^16^ Parents/guardians (hereafter referred to as “parents”) were asked whether each statement (e.g. “is helpful if someone is hurt, upset or feeling ill”) was “not true” (scored 0), “somewhat true” (scored 1), or “certainly true” (scored 2) of their child’s behaviour over the last six months. Responses were summed, with total scores ranging from 0–10. Based on Canadian-specific cut-offs,^17^ we categorized children with total scores of 8–10 as having normative levels of parent-rated prosocial behaviour (previously labeled “normal”) and those with scores of 0–7 as having nonnormative levels (previously labeled “borderline” [7] and “clinical” [0–6]). 

Happiness was measured in cycles 1, 2, 5 and 6 (2007–2011, 2016–2019) using the single-item emotion attribute measure from the Health Utilities Index Mark 3 (HUI3).^18^ Parents were asked (with input from their child if needed) whether they would describe their child as being usually “happy and interested in life,” “somewhat happy,” “somewhat unhappy,” “unhappy with little interest in life,” or “so unhappy that life is not worthwhile.” Consistent with the coding used for the youth and adult self-rated version of this measure in the PMHSIF,^1^ we categorized “happy and interested in life” as high perceived happiness.

***Analysis***

We conducted analyses using SAS Enterprise Guide Version 7.1. We used sampling and bootstrap weights from Statistics Canada to account for the sampling design. We combined cycles that measured the PMH outcome(s) of interest and combined weight files in line with Statistics Canada recommendations.^19^, ^20^ Also aligned with their recommendations,^20^ we specified degrees of freedom in our analyses based on the total number of data collection sites sampled minus the number of regions for each cycle. To examine potential differences over time, we obtained overall and sex-stratified weighted percentages with 95% confidence intervals (CIs) for normative parent-rated prosocial behaviour and high perceived happiness at each relevant cycle. We also conducted unadjusted logistic regression analyses to determine whether there were any significant differences compared to 2007–2009 (i.e. cycle 1). To investigate potential sociodemographic differences (specific variables and breakdowns are reported in Table 2), we obtained weighted percentages with 95% CIs for normative parent-rated prosocial behaviour and high perceived happiness for each group and conducted unadjusted logistic regression analyses. Similar analyses were conducted to examine how prosocial behaviour and happiness were associated. We used pairwise deletion to deal with missing data (sample sizes are reported in the notes for Tables 1 and 2.

**Table 2:** Comparing normative parent-rated prosocial behaviour and high perceived happiness across sociodemographic characteristics and to each other, children aged 6 to 11 years, CHMS, 2007–2015/2019, Canada

Sociodemographic characteristic / PMH outcome	Normative parent-rated prosocial behaviour		High perceived happiness	
	Weighted % (95% CI)	OR (95% CI)	Weighted % (95% CI)	OR (95% CI)
Sex				
Female	93.8 (92.5–95.2)	Reference	92.6 (90.8–94.5)	Reference
Male	83.3 (79.8–86.7)	**0.33 (0.23–0.46)**	90.6 (88.4–92.9)	0.77 (0.54–1.10)
Age (years)				
6–7	85.6 (81.5–89.6)	Reference	94.3 (92.2–96.4)	Reference
8–9	89.8 (87.6–92.1)	**1.49 (1.05–2.12)**	92.0 (89.2–94.8)	0.69 (0.39–1.24)
10–11	89.6 (87.3–91.9)	1.45 (0.99–2.14)	88.9 (86.4–91.4)	**0.48 (0.30–0.78)**
Household income tertile				
Low	88.4 (84.8–91.9)	Reference	90.8 (87.9–93.6)	Reference
Middle	88.4 (85.2–91.6)	1.01 (0.62–1.64)	92.7 (90.1–95.3)	1.29 (0.80–2.10)
High	88.5 (85.9–91.0)	1.01 (0.67–1.53)	91.8 (87.5–96.2)	1.14 (0.52–2.51)
Household educational attainment				
High school or less	89.2 (84.9–93.5)	Reference	89.6 (86.1–93.1)	Reference
Post-secondary	88.0 (86.1–90.0)	0.90 (0.57–1.40)	91.9 (90.1–93.7)	1.31 (0.81–2.14)
Household composition				
Lone-parent	85.7 (77.3–94.2)	Reference	86.6 (79.1–94.0)	Reference
Two-parent	88.8 (86.5–91.1)	1.32 (0.60–2.91)	92.1 (90.6–93.6)	1.81 (0.92–3.57)
Prosocial behaviour				
Nonnormative	N/A	N/A	73.6 (65.2–82.0)	Reference
Normative	N/A	N/A	94.9 (93.7–96.1)	**6.67 (4.20–10.60)**
Happiness				
Not high	58.0 (47.3–68.8)	Reference	N/A	N/A
High	90.2 (87.6–92.8)	**6.67 (4.20–10.60)**	N/A	N/A

**Source:** Canadian Health Measures Survey. 

**Abbreviations: **CI, confidence interval; OR, odds ratio; PMH, positive mental health; N/A, not applicable. 

**Notes:** All estimates are weighted. Analyses involving prosocial behaviour and sociodemographic characteristics are based on combined data from cycles 1-4 of the Canadian Health Measures Survey (degrees of freedom = 46), with sample sizes of 4196 for the sex, age, and household income tertile comparisons; 4046 for the household educational attainment comparison; and 3388 for the household composition comparison. Analyses involving happiness and sociodemographic characteristics are based on combined data from cycles 1, 2, 5, and 6 of the Canadian Health Measures Survey (degrees of freedom = 46), except for the analysis involving household income tertile that only includes cycles 1, 2, and 6 (degrees of freedom = 35). In those happiness analyses, sample sizes are 4205 for the sex and age comparisons, 4129 for the household educational attainment comparison, 3377 for the household composition comparison, and 3165 for the household income tertile comparison. The sample size was notably lower for analyses involving household composition as we excluded households with others living in the house beyond the child's parent(s) and sibling(s) (e.g. grandparent, uncle/aunt); nevertheless, interpretation of results did not change when these households were included. To account for rising household income over time, tertiles were calculated separately for each cycle. Income tertiles could not be calculated for cycle 5 as permission was not obtained from respondents for sharing household income data with Health Canada or the Public Health Agency of Canada in that cycle. Analyses examining the association between prosocial behaviour and happiness includes cycle 1 and 2, with a sample size of 2149 (degrees of freedom = 24). Odds ratios that are bolded have confidence intervals that exclude 1.0 and are considered statistically significant.

## Results

The vast majority of children had normative levels of parent-rated prosocial behaviour at each time point, ranging 86.0%–90.2% overall, 93.4%–94.6% for females and 77.9%–87.0% for males. Similarly, high perceived happiness was reported for most children, ranging 91.1%–92.2% overall, 89.4%–94.9% for females and 88.7%–93.0% for males. The prevalence of normative parent-rated prosocial behaviour and high perceived happiness in later years did not significantly differ from those observed in 2007–2009 (Table 1). 

Sociodemographic comparisons are reported in Table 2. Males had significantly lower odds of having normative parent-rated prosocial behaviour compared to females (83.3% vs. 93.8%, respectively; OR = 0.33, 95% CI: 0.23–0.46). Moreover, 8- to 9-year-olds had significantly higher odds of having normative parent-rated prosocial behaviour compared to 6- to 7-year-olds (89.8% vs. 85.6%, respectively; OR = 1.49, 95% CI: 1.05–2.12). In contrast, 10- to 11-year-olds had significantly lower odds of having high perceived happiness compared to 6- to 7-year-olds (88.9% vs. 94.3%, respectively; OR = 0.48, 95% CI: 0.30–0.78). No other significant sociodemographic differences were found.

Prosocial behaviour and happiness were positively associated (OR = 6.67, 95% CI: 4.20–10.60; Table 2). Children with high perceived happiness had significantly higher odds of having normative parent-rated prosocial behaviour (90.2%) compared to children without high perceived happiness (58.0%), and children with normative parent-rated prosocial behaviour had significantly higher odds of having high perceived happiness (94.9%) compared to children with nonnormative parent-rated prosocial behaviour (73.6%).

## Discussion

Most children in Canada had normative parent-rated prosocial behaviour and high perceived happiness, with no notable differences since 2007–2009. The high prevalence is unsurprising for normative prosocial behaviour as its cut-off was selected to include approximately 80% of the highest scores on the SDQ prosocial subscale for children and youth in CHMS cycles 3 and 4 (2012–2015).^17^ Whether normative prosocial behaviour and high perceived happiness remain as prevalent among children in more recent years could be explored if/when datasets from subsequent CHMS cycles with relevant data become available.

Examining other Canadian data,^1^ the percentages of youth and adults who reported usually being “happy and interested in life” were lower than the estimates obtained for children in this study. However, we are unable to discern whether this is due to age-related changes,^21^ generational differences, or methodological differences.^22^ For instance, parental perceptions of mental health can be more positive than self-rated perceptions.^23^ Longitudinal data are needed to provide clearer explanations. While children were present during the questionnaire administration to provide input if needed, it is unclear how much they actually contributed. The amount of convergence/divergence between parent-rated and child-rated PMH indicators could be explored in future studies. The importance of capturing children’s perspectives in the measurement and reporting of their PMH was a key takeaway from the aforementioned scoping review^3^ and was an area flagged for future research by the OECD.^24^

We found sex differences in parent-rated prosocial behaviour, with normative levels being more common among females than males. This replicates previous research on prosocial behaviour, although the size of this difference can depend on the type of prosocial behaviour, the measurement method and the target of the behaviour, and could be partially influenced by gender stereotypes.^25^ Also consistent with previous findings,^25^ the older age groups of children tended to have higher parent-rated prosocial behaviour than the youngest (although the difference for the oldest age group did not reach statistical significance; *p* = .06). Conversely, perceived happiness was highest among the youngest (vs. oldest) age group. These discrepant findings reinforce the importance of monitoring multiple PMH outcomes.^2^, ^3^, ^24^

In sum, this study furthers our understanding of the epidemiology of prosocial behaviour and happiness among Canadian children. However, there are limitations. The very high prevalence for both indicators may be indicative of ceiling effects, which could have hindered the detection of differences over time and between groups. The presence of the child and interviewer could have also contributed to the high prevalence via socially desirable responding by parents.^26^ Moreover, while the positive association between prosocial behaviour and happiness aligns with previous research^27^ and provides evidence for their convergent validity, common method biases may also contribute to some of their overlapping variance.^28^ The cross-sectional nature of the data limits causal inferences; whether perceived prosocial behaviour leads to perceived happiness (or vice versa) cannot be established. Results may not generalize to those excluded from the CHMS (e.g. those living on First Nations reserves). Ongoing surveillance beyond 2019 and of other (self-reported) PMH indicators are needed for a comprehensive understanding of child PMH in Canada. Nevertheless, this study provides initial insights into the PMH of children in Canada based on two indicators, which could be integrated into surveillance tools like the PMHSIF. 

## Conflicts of interest

The authors have no conflicts of interest to disclose.

## Authors’ contributions and statement

CAC: Conceptualization, methodology, formal analysis, writing – original draft.

DL:Writing – review and editing.

KRA: Writing – review and editing.

LLO: Writing – review and editing.

MV: Writing – review and editing, validation.

The content and views expressed in this article are those of the authors and do not necessarily reflect those of the Government of Canada.

## References

[B01] Positive Mental Health Surveillance Indicator Framework: overview [Internet]. PHAC.

[B02] Orpana H, Vachon J, Dykxhoorn J, McRae L, Jayaraman G (2016). Monitoring positive mental health and its determinants in Canada: the development of the Positive Mental Health Surveillance Indicator Framework. Health Promot Chronic Dis Prev Can.

[B03] Varin M, Jhumi M, Capaldi CA, Dopko RL, Ooi LL Positive mental health among children 11 years and under in Western countries: a scoping review to inform Canada’s public health surveillance. medRxiv [Preprint].

[B04] Dunfield KA (2014). A construct divided: prosocial behavior as helping, sharing, and comforting subtypes. Front Psychol.

[B05] Denham SA, Brown C (2010). “Plays nice with others”: social–emotional learning and academic success. Early Educ Dev.

[B06] Huber L, tner M, Schmitz J (2019). Social competence and psychopathology in early childhood: a systematic review. Eur Child Adolesc Psy.

[B07] Oishi S, Graham J, Kesebir S, Galinha IC, Psychol B (2013). Concepts of happiness across time and cultures. Pers Soc Psychol B.

[B08] Huta V, Waterman AS (2014). Eudaimonia and its distinction from hedonia: developing a classification and terminology for understanding conceptual and operational definitions. J Happiness Stud.

[B09] Canadian Health Measures Survey (CHMS): detailed information for spring 2007 to spring 2009 (cycle 1) [Internet]. Statistics Canada.

[B10] Canadian Health Measures Survey (CHMS): detailed information for August 2009 to November 2011 (cycle 2) [Internet]. Statistics Canada.

[B11] Canadian Health Measures Survey (CHMS): detailed information for January 2012 to December 2013 (cycle 3) [Internet]. Statistics Canada.

[B12] Canadian Health Measures Survey (CHMS): detailed information for January 2014 to December 2015 (cycle 4) [Internet]. Statistics Canada.

[B13] Canadian Health Measures Survey (CHMS): detailed information for January 2016 to December 2017 (cycle 5) [Internet]. Statistics Canada.

[B14] Canadian Health Measures Survey (CHMS): detailed information for January 2018 to December 2019 (cycle 6) [Internet]. Statistics Canada.

[B15] Goodman R (1997). The Strengths and Difficulties Questionnaire: a research note. J Child Psychol Psyc.

[B16] Hoffmann MD, Lang JJ, Guerrero MD, Cameron JD, Goldfield GS, Orpana HM, et al (2020). Evaluating the psychometric properties of the parent-rated Strengths and Difficulties Questionnaire in a nationally representative sample of Canadian children and adolescents aged 6 to 17 years. Health Rep.

[B17] Turner SE, Dopko RL, Goldfield G, Cloutier P, Pajer K, Abdessemed M, et al (2023). Validating existing clinical cut-points for the parent-reported Strengths and Difficulties Questionnaire in a large sample of Canadian children and youth. Health Promot Chronic Dis Prev Can.

[B18] Horsman J, Furlong W, Feeny D, Torrance G (2003). The Health Utilities Index (HUI): concepts, measurement properties and applications. Health Qual Life Out.

[B19] (2019). Combining weights – instructions: Canadian Health Measures Survey (CHMS). Statistics Canada.

[B20] (2021). Instructions for combining multiple cycles of Canadian Health Measures Survey (CHMS) data. Statistics Canada.

[B21] Buecker S, Luhmann M, Haehner P, hler JL, Dapp LC, Luciano EC, et al (2023). The development of subjective well-being across the life span: a meta-analytic review of longitudinal studies. Psychol Bull.

[B22] Khanna D, Khadka J, Mpundu-Kaambwa C, Lay K, Russo R, Ratcliffe J (2022). Are we agreed. Pharmacoeconomics.

[B23] Canadian Health Survey on Children and Youth, 2019 [Internet]. Statistics Canada.

[B24] (2021). Measuring what matters for child well-being and policies. OECD Publishing.

[B25] Eisenberg N, Fabes RA, Spinrad TL, Eisenberg N, Damon W, Lerner RM, th ed (2007). Prosocial development. Handbook of child psychology.

[B26] Zhang X, Kuchinke L, Woud ML, Velten J, Margraf J (2017). Survey method matters: online/offline questionnaires and face-to-face or telephone interviews differ. Comput Hum Behav.

[B27] Aknin LB, Vondervoort JW, Hamlin JK (2018). Positive feelings reward and promote prosocial behavior. Curr Opin Psychol.

[B28] Podsakoff PM, MacKenzie SB, Podsakoff NP (2003). Common method biases in behavioral research: a critical review of the literature and recommended remedies. J Appl Psychol.

